# Calcific Aortic Stenosis—A Review on Acquired Mechanisms of the Disease and Treatments

**DOI:** 10.3389/fcvm.2021.734175

**Published:** 2021-09-17

**Authors:** Banafsheh Zebhi, Mohamad Lazkani, David Bark

**Affiliations:** ^1^Department of Mechanical Engineering, Colorado State University, Fort Collins, CO, United States; ^2^Medical Center of the Rockies, University of Colorado Health, Loveland, CO, United States; ^3^Department of Pediatrics, Washington University in Saint Louis, Saint Louis, MO, United States; ^4^Department of Biomedical Engineering, Washington University in Saint Louis, Saint Louis, MO, United States

**Keywords:** calcific aortic stenosis, prosthetic heart valve, basilica, transcatheter heart valve, hemodynamics, coronary obstruction

## Abstract

Calcific aortic stenosis is a progressive disease that has become more prevalent in recent decades. Despite advances in research to uncover underlying biomechanisms, and development of new generations of prosthetic valves and replacement techniques, management of calcific aortic stenosis still comes with unresolved complications. In this review, we highlight underlying molecular mechanisms of acquired aortic stenosis calcification in relation to hemodynamics, complications related to the disease, diagnostic methods, and evolving treatment practices for calcific aortic stenosis.

## Introduction

Calcific aortic stenosis (AS) is the most common valve disease in developed countries (1, 2), in which valves thicken and stiffen, and in some cases nodular deposits form, limiting valve function. This may result in valve regurgitation with concomitant stenosis. Calcific AS is a progressive disease that advances with age (3, 4), affecting ~0.2% of people 50–59 years of age and increasing to 9.8% for 80–89 years (5, 6). As the general population has become older, the prevalence of calcific AS has increased, igniting multiple improvements in its management (2). In addition to new diagnostic imaging techniques emerging, novel prosthetic valves have been developed as an effective treatment for calcific AS. To date, pharmacotherapy has not been shown to slow down the progression of the disease, or to reverse the calcification process (2). In this review we highlight engineering perspectives toward recent advancements in the treatment of AS, underlying molecular pathways and mechanisms of the calcification process, clinical characteristics, hemodynamics, complications of calcific AS, diagnoses, and common treatment practices for calcific AS.

## Aortic Valve Structure and Calcification

**Figure 1:**
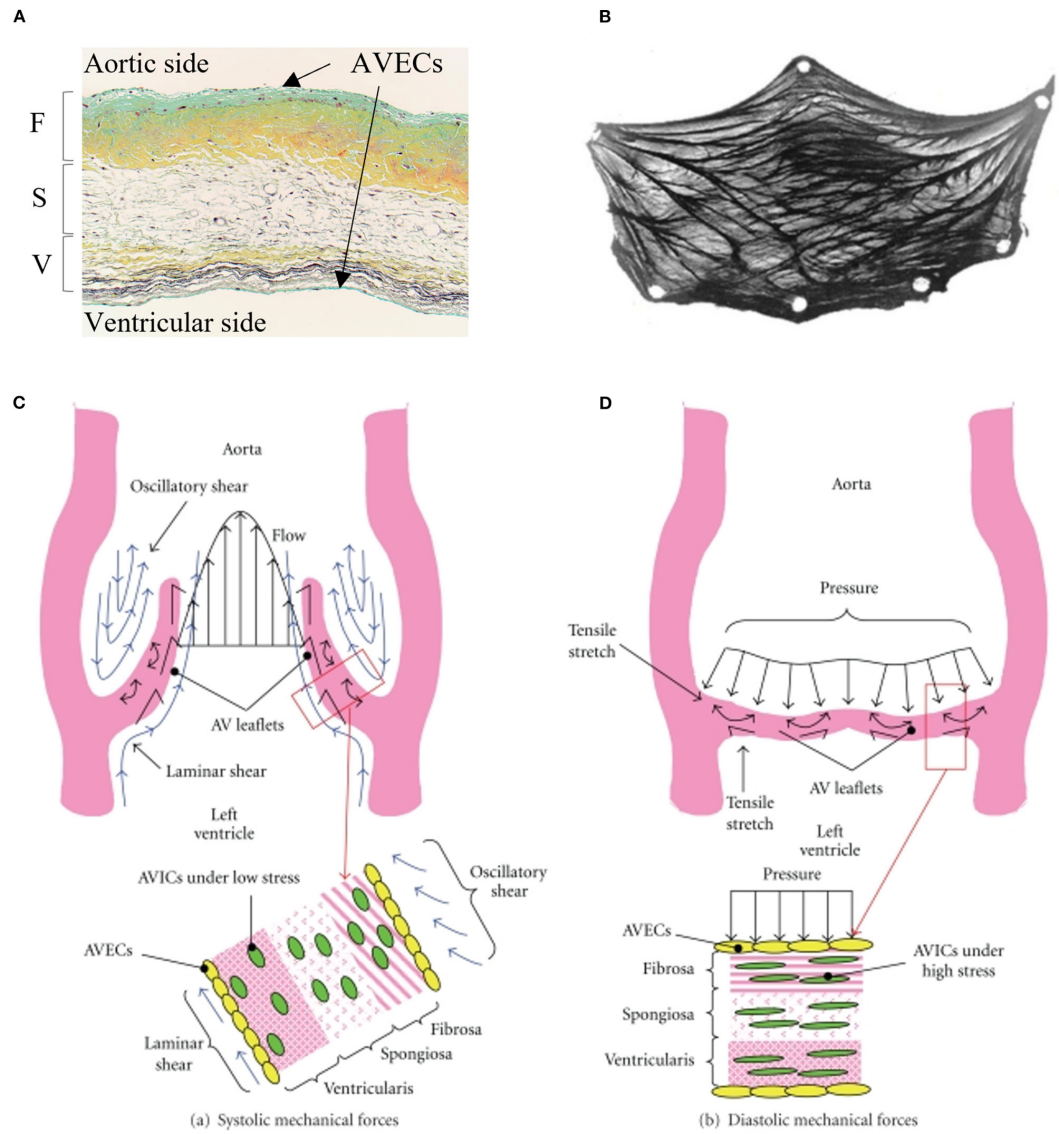
Microscopic and macroscopic overview of aortic valve tissue structure: **(A)** histological section of the aortic valve leaflet showing three layers: fibrosa (F), spongiosa (S), and ventricularis (V) covered by valvular interstitial cells (VICs) and valvular endothelial cells (VlvECs). **(B)** excised view of the aortic valve leaflet demonstrating fiber structure. Schematic of stress experienced by aortic valve leaflets and valvular cells during **(C)** systole, and **(D)** diastole. **(A)** is from Fishbein et al. **(B)** is from Driessen et al. **(C,D)** are from Balachandran et al. with permission.

Aortic valve (AV) leaflets consist of three layers: the ventricularis layer is elastin-rich and located on the ventricular side; the spongiosa is made of proteoglycans that provide lubrication for the other layers; and a fibrosa layer made of a dense collagen network is on the aortic side of the valve (7, 8), which provides much of the structural support in response to mechanical forces (9). These 3 layers are filled with valvular interstitial cells (VICs), and the entire layered structure is covered by endothelial cells (10) (Figure 1). The fibrosa layer is particularly prone to calcification (11), while alterations to the endothelial barrier function could impact propensity for calcification. For years, calcification was thought to be a passive degenerative process in which calcium accumulates on leaflets (4, 12–17), where old age, male gender, diabetes mellitus, coronary artery disease, chronic renal disease, hypertension, hypercholesterolemia, and smoking are known to increase the risk for AV calcification (18). Now, it is understood that calcification is a complex process involving mechanobiology, molecular signaling, tissue remodeling, and inflammation as the AV opens and closes billions of times during a lifetime.

## Hemodynamics and Endothelial Cell Mechanotransduction

Due to the sensitivity to hemodynamics (blood flow), endothelial cells may contribute to calcification and AS by responding to shear stress experienced on the cells' apical side (Figure 1). Indicating a potential link, calcium formation is more common in the non-coronary cusp, where surrounding fluid wall shear stress is lower relative to coronary cusps (1). Endothelial cells respond to shear stress by changing their morphology, gene regulation, protein expression, transendothelial transport, alignment, and release of molecules and proteins from the surface (19). These processes can occur as endothelial cells convert mechanical stimuli to biochemical signals to elicit biological responses, known as mechanotransduction, briefly summarized below.

Vascular endothelial cells sense their environment through ion channels (which allows membrane depolarization and cell signaling), integrins, intercellular junction proteins, caveolae, the glycocalyx, G protein-coupled receptors (GPCRs), and tyrosine kinase receptors (20, 21), However, only some of these mechanosensors have been observed for valvular endothelial cells (further explained below). Integrins function as signaling receptors and play a crucial role in transmitting physical mechanical forces between the extracellular matrix and the actin cytoskeleton via focal adhesion complexes. In one example, valvular endothelial cell morphological alignment perpendicular to the direction of flow involves β1 integrin, vinculin and focal adhesion kinase and depends on Rho-kinase and calpain (19). GPCRs are also highly sensitive to changes in flow and activate downstream signaling by binding to extracellular ligands (22). The glycocalyx is a mediator for cell-cell adhesion and works as a trap for ions and antibodies that translate to downstream signaling pathways (20). Using these mechanosensors (and others), mechanical forces are transmitted to the nucleus and can change the nuclear morphology, stiffness, and gene expression (23). Mechanotransduction in relation to AS calcification continues to be explored and only a brief description of some findings are presented here.

## Inflammation Mechanism in Aortic Valve Calcification

Multiple studies indicate a role for an innate and adaptive immune response that leads to calcification. This largely initiates with dysregulated valvular endothelial cells, progresses to excessive remodeling of the leaflet ECM, changes in tissue stiffness, tissue mineralization, osteogenesis (formation of bone), and eventually lead to late-stage calcification (24, 25).

The endothelium is most responsive to the magnitude and directionality of fluid shear stress. Physiological unidirectional shear stress is protective by downregulating adhesion proteins, vascular cell adhesion molecule 1 (VCAM-1), platelet endothelial cell adhesion molecule-1 (PECAM-1), and chemokines IL-1β and IL-8. It also leads to expression of nitric oxide (NO), which can help prevent thrombotic responses that could otherwise play a role in calcification (8). Notch signaling is increased, which helps prevent calcification (26, 27). There is also increased expression of osteoprotegrin (OPG), which regulates aortic valve calcification by inhibiting receptor activator of the nuclear factor κB ligand (RANKL) signaling (28, 29). Under oscillatory shear stress, VCAM-1, intercellular adhesion molecule 1 (ICAM-1), endothelial selectin (E-selectin), VEGF, and TGFβ are upregulated, which leads to increased oxidative stress and inflammatory agents such as bone morphogenic protein (BMP)-4 and cytokines: IL-1β and INFγ. TGFβ and VEGF can induce cell proliferation, fibrosis, and promotes calcification by enhancing irreversible tissue thickening and stiffening (19). Increased BMP-2 and BMP-4 can upregulate osteogenic pathways involving the Msx2 transcription factor that activates Wnt/LDL receptor-related protein 5 (Lrp5)/β-catenin signaling (30, 31), and the Runx2/Cbfa1 transcription factor (31, 32) that leads to differentiation of the VICs to an osteoblast-like phenotype. Altogether, low and oscillatory shear stress found in stagnating regions of aortic valve leaflets are linked to signaling changes in the endothelium that lead to proinflammatory responses that could be linked to calcification.

Endothelial cell responses can also lead to low-density lipoprotein (LDL) deposition in response to altered mechanical forces, which can induce inflammation (25, 33). LDL and lipoprotein (a) derived from cholesterol colocalize in the calcified valve tissue in early calcification (34). Plasma lipoprotein (a) is an independent risk factor of AS identified through genome-wide association studies (34–36). Furthermore, apolipoprotein H (APOH) was identified as a novel locus for lipoprotein (a) levels (36). Despite the link of LDL with calcification, studies have found that LDL suppression or lipid-lowering therapy with statins (anti-inflammatory and antioxidant agents) do not slow down the progression of disease even when given at early stages of calcification (37–41).

**Figure 2:**
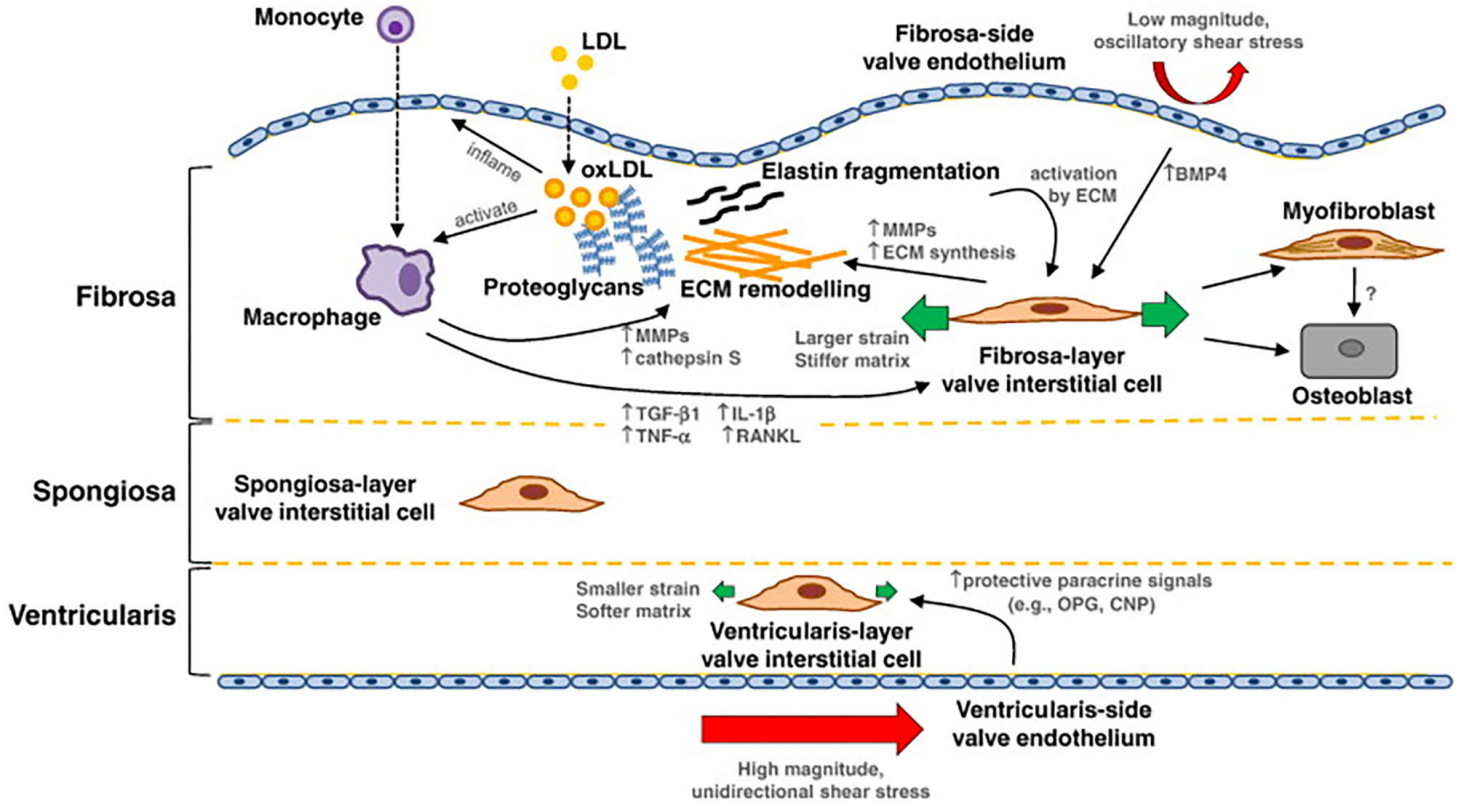
Summary of mechanotransduction and pathway of valvular calcification: in the fibrosa layer, the oxidated LDL (oxLDL) can inflame the endothelial cells, bind to monocytes, and activate macrophages. Activated macrophages mediate extracellular matrix (ECM) remodeling and molecular signaling that can potentiate valvular interstitial cell (VIC) pathological differentiation to myofibroblast and osteoblast cells. The ECM further affects VIC activation and differentiation; activated VICs synthesize and remodel the ECM, and produce cytokines, like TGF-β1. Interstitial and endothelial cells on each layer of the tissue exhibit a different phenotype. On the ventricular side, endothelial cells experience high magnitude and unidirectional shear stress, which may inhibit pathological differentiation of the local VICs. Image is from Yip et al. with permission.

Macrophages are found in calcified AV leaflets, likely entering through trans-endothelial migration involving ICAM and VCAM (37). In response to activated endothelial cells, macrophages release pro-osteogenic cytokines like IL-1β, IL-6, tumor necrosis factor-α (TNF-α), and RANKL, all of which could contribute to calcification. Activated macrophages produce enzymes that can cause interstitial cell activation, changes in gene expression, and differentiation to osteoblasts, which then leads to excess synthesis and remodeling of collagen fibers in the fibrosa (11) (Figure 2). Cytokines can promote cell proliferation and ECM remodeling. Some fibroblasts can differentiate to activated myofibroblasts (42). The activation of myofibroblasts further induces inflammation through the expression of BMP, MMP-2, and MMP-9 and releases TNF-α and TGF-β1 and eventually differentiate to osteoblast-like phenotype (43). TNF-α activates nuclear factor-κB (NF-κB) pathways which leads to expression of proinflammatory genes (44–46). Via activation of NF-κB, T cell activation amplifies the inflammatory response by producing cytokine interferon- (IFN-γ) and TNF-α. Macrophages (along with vascular smooth muscle cells) also release calcification-prone extracellular vesicles (EVs) (47, 48). Excessive production of EVs lead to microcalcification. Overall, macrophages can initiate a number of proinflammatory events that can lead to calcification in response to endothelial signals.

## Pharmacotherapies

Currently there is no approved pharmaceutical treatment for calcific aortic valve stenosis, but literature provides possible future pharmacological approaches in human and animal models. A review by Myasoedova et al. (49) showed that oxidized low density lipoprotein (Ox-LDL), oxidized phospholipids (Ox-PL), lipoprotein associated phospholipase A2 (Lp-PLA2), Lp(a), proprotein convertase subtilisin/kexin type 9 (PCSK9), high density lipoprotein (HDL), the purinergic receptor 2Y2 (P2Y2R), sodium-dependent phosphate cotransporter (PiT-1), dipeptidyl peptidase-4 (DDP-4) are targetable components for prevention and treatment of calcific AS in human. In efforts to target calcific AS, antisense oligonucleotides (ASOs) 2nd generation [inhibitor of apo(a) mRNA translation] was introduced as a new selective Lp(a) inhibitor (50, 51). Niacin (nicotinic acid) therapy helps to lower LDL and Lp(a) (52) and increase HDL (53). Since statins exhibit limited benefit to calcific AS, the lowering of LDL may not provide benefit. Also, a trial study showed that extended-release niacin (ERN) does not reduce the risk of cardiovascular disease despite the favorable effect on lowering Lp(a) (54). PCSK9 (involve in regulating blood cholesterol) inhibitors can significantly lower LDL and plasma Lp(a) (55, 56) and reduce the risk of cardiovascular disease, but have an unclear impact on calcific AS. Sodium phosphonoformate (PFA) as a PiT-1 inhibitor can inhibit calcification in human VICs (57). DDP-4 inhibitors inhibit progression of calcific AS by blocking insulin-like growth factors and osteogenic activities in VICs. Additionally, some animal studies suggest that calcification can be reversible. Miller et al. (58) showed that a “genetic switch” in Reversa mice can reduce plasma lipid and oxidative stress and halt the progression of the calcific AS. P2Y2R promotes expression of carbonic anhydrase CAXII, which acidifies the extracellular space and promotes calcification regression by resorbing minerals in mice (59). There is ongoing effort to develop pharmacotherapies for calcific AS, but due to the complex processes involved, this is a challenging undertaking.

## Clinical and Hemodynamic Characteristics of Aortic Stenosis

Severe AS can result in serious problems. Patients can experience heart murmur, chest pain, shortness of breath, fatigue and syncope. Pressure overload can occur in the left ventricle, and when left untreated, this can lead to hypertrophy (60). Presence of long-term pressure overload can even eventually lead to systolic failure and congestive heart failure. AS can also further create bleeding complications described below.

Aortic stenosis severity can be assessed based on valve flow velocity, valve orifice area, and the pressure gradient across the valve (61, 62). The common flow condition for severe stenosis is defined as a peak aortic velocity ≥ 4 (m/s), pressure gradient ≥40 (mmHg), and AV area <1 (cm^2^) (63); however, 5–10% of the patients with severe stenosis have low flow (low cardiac output), low pressure gradient <40 (mmHg) due to reduced left ventricle ejection fraction (LVEF) (<40%) (62), and 10–35% with severe stenosis have paradoxical (Stage 3D Severe AS) low flow and low pressure gradient due to LV hypertrophy (with normal EF). These variations of hemodynamics make the diagnosis and decision making for treatment of AS difficult; therefore other parameters have also been used to make accurate decisions when treating AS; this includes both subjective clinical symptoms and objective data, such as valvulateral impedance, AV resistance, projected AV area at normal flow, and calcium score (62, 63). A review by Saikrishnan et al. (62) provides a comprehensive summary of metrics, units, methods of measurement and the cut-off points for severe AS. In order to score AS, maximum velocity and pressure gradient are measured, and valve effective orifice area (EOA) is calculated.

**Figure 3:**
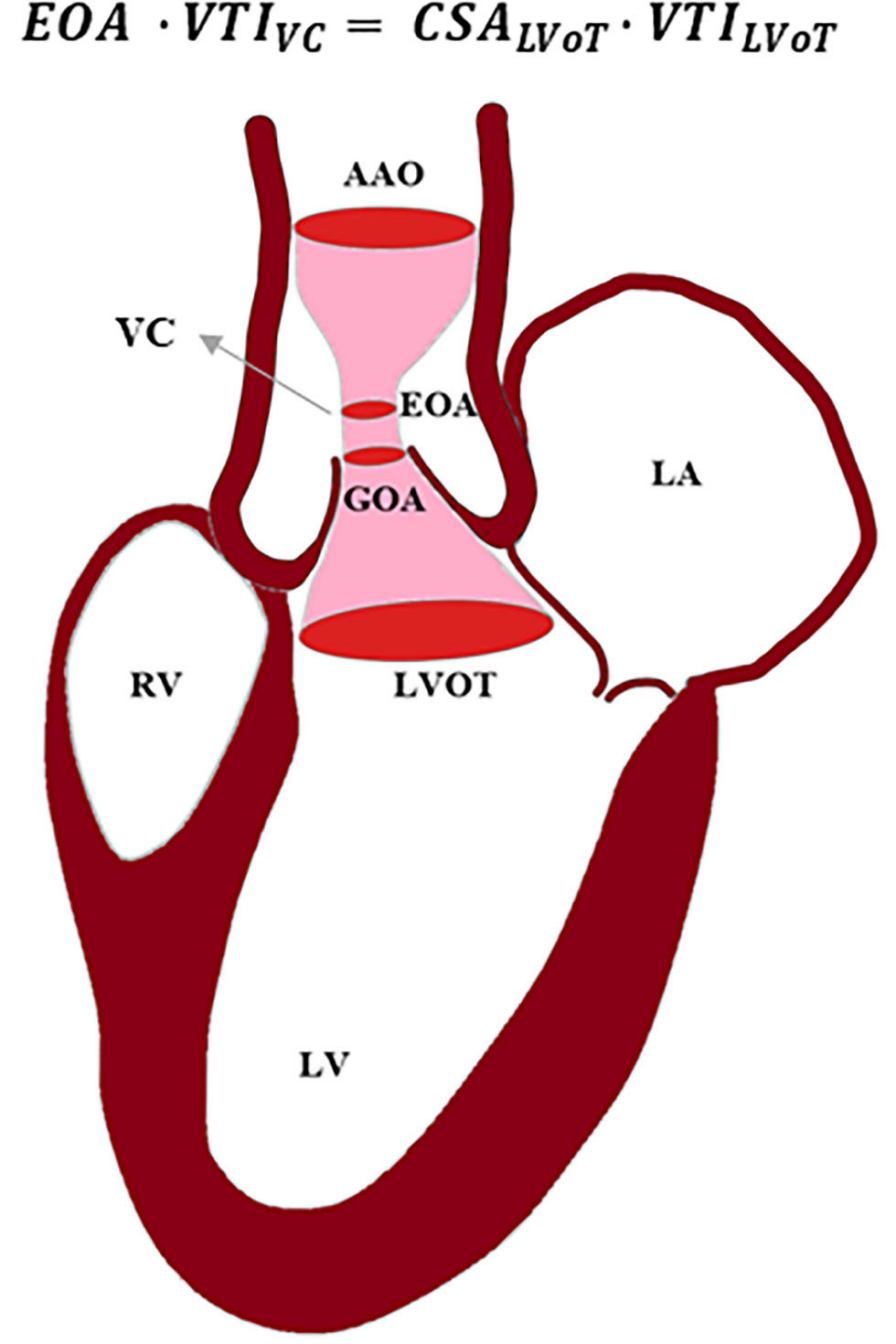
Schematic of blood passing through a stenosed aortic valve. Using continuity equation, the effective orifice area (EOA) can be calculated based on velocity time integral (VTI) at vena contracta (VC), cross-sectional area (CSA) of left ventricle outflow tract (LVOT), and VTI at LVOT. AAO, ascending aorta; LA, left atrium; LV, left ventricle; RV, right ventricle; GOA, geometric orifice area.

Blood flow through the valve can be characterized using techniques and imaging modalities described below. Blood flowing from left ventricle outflow tract (LVOT), passing through a stiff narrow valve opening, creates a jet with maximum velocity at vena contracta (VC). VC is a location where fluid pathlines converge, and the velocity is the highest. The area of the VC is known as the EOA. Using Doppler echocardiography, pressure drop is approximated using a simplified Bernoulli equation, assuming that proximal velocity is negligible, Δ*P* = 4*v*^2^, where Δ*P* is the transaortic valve pressure gradient (between VC and LVOT), and *v* is maximum velocity of blood (64) (Figure 3). EOA is calculated using the continuity equation; the volume flow rate passing through LVOT equals to the flow rate passing through VC, i.e., *EOA* · *VTI*_*VC*_ = *CSA*_*LVoT*_ · *VTI*_*LVoT*_, where *VTI*_*VC*_ and *VTI*_*LVoT*_ are the velocity time integrals measured from the parasternal long-axis view at the location of LVOT and VC, and *EOA* and *CSA*_*LVoT*_ are cross sectional areas of VC and LVOT (62, 64, 65). In rare cases when there are discrepancies in Doppler echocardiography measurements, cardiac catheterization is used to obtain a more accurate measurement of pressure directly from the blood vessel. Using the Gorlin equation, the geometric orifice area (GOA) is calculated from the flow rate and the pressure drop between the LVOT and VC, which is related to the EOA through the contraction coefficient. The GOA the area formed by free edges of the leaflets when valves are fully opened. Thus, catheterization measurements are performed at peak systole. A review by Saikrishnan et al. (62) provides a detailed description of diagnostic modalities and formulations. Calcific AS can be additionally assessed by computed tomography (CT) which provides high-resolution assessment of calcification, and enables accurate measurement of leaflet anatomy and annulus geometry. Calcific deposits have higher density compared with surrounding soft tissues. CT imaging uses attenuation coefficients expressed by Hounsfield unit (HU). High density calcific deposits have a high attenuation value (>130 HU) which makes the calcific area appear bright in the image. A calcium score is quantified by multiplying calcified area by Hounsfield unit, and is known as Agatston score (66, 67). Different Agatston scores are used for men and women to diagnose severe stenosis (68–70). Recent studies highlighted that calcification deposits are more prevalent in men, while fibrosis may be more significantly involved in valvular dysfunction in women (71–73); presence of estrogen in women inhibits aortic valve calcification via suppression of RANKL signaling (74, 75) and suppression of TGFβ-dependent ECM production (76). Animal studies showed that sex-related differences in calcific aortic valve disease are due to different pathogenetic and signaling pathways in male and female (72).

In addition to impacting energy loss and hemodynamics, an aortic stenosis has a significant impact on the hemostatic capacity of blood. It can lead to gastrointestinal, skin, and mucosal bleeding, which may, in-part, be attributed to acquired von Willebrand syndrome (AVWS) also known as Heydes Syndrome (77–81). It appears as though the AVWS stems from turbulence that can occur in an aortic stenosis, whereas it is often alleviated once a diseased valve is replaced, eliminating pathological flow (78, 82–86).

## Calcific Aortic Valve Stenosis Treatment

At late stages of calcific AS, no therapies can manage the progression of calcification and the only effective treatment is valve repair or replacement (61).

### Valve Repair

Valve repair surgery can be used and is one of the oldest cardiovascular surgical interventions dating back to the early 1920s (87). Native aortic valve (root and leaflets) repair comes with low mortality risk and is free of most valve-related complications, yet durability of treatments remained limited and reoperation is often required in the short term (88). This has largely fallen out of favor in modern practice and is not utilized often, except in some centers.

### Valvuloplasty

Balloon aortic valvuloplasty (BAV) is a catheter-based technique that dilates native valve's narrowed opening by delivering and inflating a balloon at the site of stenosed valve through femoral artery (89). BAV increases leaflet mobility by creating a fracture in calcified lesions, expanding the aortic annulus and separating calcified commissures (90). It has become a tool that can even be used in fetal aortic stenosis, to avoid progression into a more complex congenital heart malformation (91–94). Use of an oversized balloon can cause infractions in the valve ring, separation between leaflets and the root, and leaflet tearing (90). Additionally, balloon inflation may cause complications like coronary ostia occlusion that could lead to myocardial ischemia and dysfunction of left ventricle. BAV procedures do not provide long term improvements in adults, as the dilated valve can become restenosed; therefore, BAV is a temporary improvement option and a bridge to SAVR or TAVR for patients who are at high risk and need an urgent intervention (95). Utilization of BAV is also practiced as a palliative treatment option in terminal patients with <1-year life expectancy to improve quality of life in the short term, often seen in the hospice population.

### Valve Replacement

Surgical aortic valve replacement (SAVR) has become the most common treatment for severe calcific aortic stenosis in which patients undergo an open-heart surgery to replace their aortic valve with a mechanical or a bioprosthetic valve; in this procedure calcified native leaflets are cut and removed. The mechanical or bioprosthetic valve is subsequently sutured to the aortic root. SAVR improves symptoms and survival, but it comes with risks of thrombosis in mechanical valves that can cause stroke or heart attack, or in the case of bioprosthetic valves, durability is an issue with these valves often calcifying over time (96). Initially, in older patients who are inoperable or are at high risk for surgery, transcatheter aortic valve replacement (TAVR) was an alternative option. However, this option is now common practice for lower risk patients, as the devices and procedures have advanced with equal to improved outcomes compared to SAVR (97, 98). The first in-human TAVR was performed in 2002 (99); since then, more than 50,000 TAVR interventions have been done worldwide (100). TAVR is a less invasive technology in which a stented valve is delivered to the location of native valve through a catheter and is expanded to replace the calcified native aortic valve and leaflets.

Current guidelines set by the American College of Cardiology (ACC) and American Heart Association (AHA) advocate for Aortic Valve Replacement in the setting of symptomatic severe aortic stenosis. Timing of intervention depends on the development of clinical symptoms once the valve is classified as severe. The main reason is due to durability of bioprosthetic valves. Due to the relative development of TAVR being in its infancy within the last decade, long term durability has not been well-established, although expert consensus agree 10 years is a reasonable time frame before expected degeneration and failure of the bioprosthesis. However, investigators are currently attempting to determine the benefit of treatment of severe aortic stenosis before the development of symptoms and potentially remodeling and other stressful changes to the heart. An ongoing study titled Evaluation of Transcatheter Aortic Valve Replacement Compared to Surveillance for Patients With Asymptomatic Severe Aortic Stenosis (EARLY TAVR) trial is ongoing to address the timing of intervention in severe aortic stenosis (101–104). Furthermore, there is another school of thought that goes beyond waiting for symptoms with severe AS, but in fact challenges the traditional belief to only treat severe AS. A clinical trial is being developed, called PROGRESS: Management of Moderate Aortic Stenosis by Clinical Surveillance or TAVR. As such, investigators are now looking to examine the benefit of treating moderate AS with TAVR intervention, although facing the same challenges regarding the issue of durability.

SAVR and TAVR have various advantages. A study of 699 high-risk patients with severe aortic stenosis who were randomly treated with SAVR and TAVR in PARTNER 1 trial showed that 1-year mortality rates were similar between the transcatheter and surgically treated groups (24.2% TAVR vs. 26.8% SAVR), but hemodynamics and post-operative outcomes were significantly different. The transcatheter group had a shorter hospitalization with a slightly better mean AV pressure gradient and mean AV area at 1-year. However, vascular complications were significantly higher in the transcatheter group at 1-month (11% TAVR vs. 3.2% SAVR). The rate of major strokes at 1-year were more than twice as high in the transcatheter group (5.1% TAVR vs. 2.4% SAVR). Moderate and severe paravalvular regurgitation was more frequent in the transcatheter group than in the surgical group at 1-year (6.8% TAVR vs. 1.9% SAVR). Meanwhile, major bleeding was more frequent in the surgical group (19.5% SAVR vs. 9.3% TAVR) (105). Other follow-up studies have confirmed similar mortality rates and post-procedural outcomes; at 5 years, Gleason et al. (106) reported mortality rates of 55.3 and 55.4% for TAVR and SAVR, respectively, and Mack et al. (107) reported that risk of death at 5 years increases to 67.8% in TAVR and 62.4% in SAVR.

In low-risk patients, with severe aortic stenosis that were randomly treated with SAVR and TAVR in PARTNER 3 trial, TAVR was associated with significantly lower risk of mortality at 1 year (2.1% TAVR vs. 3.5% SAVR) and life threatening bleeding (3.9% TAVR vs. 11.2% SAVR); no significant differences in stroke (3.0% TAVR vs. 4.2% SAVR), major vascular complications (3.6% TAVR vs. 2.4% SAVR), and myocardial infarction (1.7% TAVR vs. 2.1% SAVR); and significantly higher moderate to severe paravalvular leak (PVL) (3.6% TAVR vs. 1.7% SAVR) (108). With 3 trials (PARTNER 1, 2, and 3), TAVR vs. SAVR have been studied in high-, intermediate-, and low-risk patients.

The TAVR utilization among underserved and underrepresented populations are lower. This was initially thought to be related to lower incident of AS among Black and Hispanic populations (109), but further studies suggested that this might be due to limited accesses to care, low socioeconomic status, and treatment biases in the non-White population (109–112). This calls the need for advance clinical care accessible to all patients regardless of their race and ethnicity.

## Mechanical Heart valves

Currently implanted mechanical heart valves (MHVs) typically have a bileaflet structure in shape of two disks made of pyrolytic carbon that can open pivotally. MHVs are highly durable when compared with other artificial heart valves; they can last up to 25 years in patients without major complications, but they have high risk of thrombosis (96). High durability makes these valves more suitable for patients younger than age 50, as MHVs have a lower risk of reoperation (113).

Fluid high shear stress in the hinge region of these valves can initiate thrombotic events (114–117). Patients treated with mechanical valves need a lifelong anticoagulant drug therapy to prevent thrombosis and thromboembolism (118); however these drugs increase the risk of bleeding, stroke, systemic embolism, cardiac tamponade and death (119). Therefore, multiple groups are attempting to improve the blood-material interactions through surface treatments (120–123). However, the hemodynamic impact on blood from the hinge remains a concern, even with these treatments.

## Bioprosthetic Heart Valves

Bioprosthetic heart valves (BHV) are made of porcine or bovine pericardium. They have the advantage of being less thrombotic, requiring only short-term anticoagulation after surgery. The main disadvantage of BHVs is that they often require reoperation due to structural valve deterioration and calcification, making the average BHV lifetime only ~15 years. In recent years, BHVs durability has been improved by anti-calcification and anti-mineralization treatments. Therefore, nowadays BHVs are more commonly recommended for implantation, even in younger patients, due to their improved durability and lower risk of structural deterioration (124). Otherwise, pediatric patients previously exhibited severe complications with calcification of BHVs.

## Transcatheter Heart Valves

Transcatheter heart valves (THVs) are gaining traction due to novel designs and delivery systems to replace the calcified aortic valve. In TAVR procedures, TAVs are deployed to the location of a calcified aortic valve with stent expansion through one of two main mechanisms: balloon expansion or self-expansion through shape memory alloys. The stent permanently opens the native valve by pushing against calcified leaflets. Some of the most frequent complications occurring with TAVR procedures are TAV malpositioning, coronary obstruction, paravalvular leak, crimped-induced leaflet damage, thrombosis, conduction abnormalities, and prosthetic-patient mismatch (125). There are also less common, but potentially fatal complications including valve embolization and annular rupture. TAV crimping causes significant structural changes and damages in leaflet tissue that affects the durability of the tissue, and can lead to early thrombosis, early calcification and endocarditis in tissue (126). Prosthetic-patient mismatch is a condition in which EOA of the TAV is too small relative to patient's body size (127) causing elevated flow resistance at the valve which should be overcome by increased pressure in the heart (125).

Valve positioning has an important role in TAV hemodynamics; it has been suggested that TAV be positioned about 5 mm below the annulus of the valve for the best outcome (128); however, the deployment site is dependent on the type of the TAV and in recent years, many attempts have been made to customize TAV deployment according to the patient-specific aortic root anatomy. If the implant is too-high or a too-low, it can result in moderate to severe paravalvular aortic regurgitation (AR) or PVL (128). The malpositioned TAV can be manually repositioned; if ineffective, an alternative solution is to deploy a second TAV inside the first TAV, this is known as valve-in-valve (ViV) procedure (128). Using new generation of TAVR devices, ViV has shown to be very effective in reducing post-procedural AR; a study of 63 patient who had ViV procedure using Edwards Sapien transcatheter valve showed that only 7.9% of the patient still had significant AR after procedure, however, ViV is associated with higher prevalence of cardiac conduction abnormalities which requires permanent pacemaker implantation in patients (129).

Additionally, undersizing a TAV can lead to malpositioning, valve dislodgement, and embolization (130). It has been recommended that slightly oversizing the TAV can minimize PVL without causing injury and rupture in aortic root and annulus (130–134). Another cause of PVL after TAVR procedure for calcified AS is the gap between the TAV and soft tissue resulting from stiffened calcified native leaflets and a calcified annulus (135). The new generation of TAVR devices are designed to reduce some of these complications. Edwards Sapien family of valves are balloon expandable TAVs comprised of a cobalt-chromium frame; an inner and an outer sealing skirts made from polyethylene terephthalate (PET) fabric to reduce PVL; and bovine pericardial leaflet tissue treated with anticalcification treatment, ThermaFix, to reduce mineralization. In contrast, the Medtronic CoreValve family are self-expandable, owing to nitinol stent material, and are comprised of porcine pericardial leaflet tissue with an antimineralization treatment. Numerous studies have investigated performance of Medtronic CoreValve and Edwards Sapien valves with respect to postprocedural PVL. Some reported that moderate to severe post-procedural PVL is more common with Medtronic CoreValve (136–138). However, a recent longitudinal study showed that the severity and frequency of PVL at pre-discharge was significantly higher in Medtronic CoreValve (56.7% Medtronic CoreValve vs. 43.2% Edwrads Sapien, *p* = 0.06), but after 1 year, there was no major differences in frequency and severity of PVL between the two groups, possibly due to coaptation of self-expandable nitinol stent with aortic annulus (139).

Conduction abnormalities can be caused by tissue damage during valve deployment. In general, balloon-expandable valves have lower rates of pacemaker requirements compared to self-expandable TAVs. Studies show that the risk of conduction abnormalities and the need for permanent pacemaker implantation is higher after Medtronic CoreValve implantation compared to Edwards Sapien (140–143), possibly due to the valve design and its self-expansion mechanism; Medtronic CoreValves have a higher height and are implanted deeper into the LVOT. The self-expandable nitinol stent may apply pressure on and below the annulus that could result in atrioventricular node and left bundle branches damage (143, 144).

## Coronary Obstruction

Surgical bioprosthetic valves are likely to degenerate within 10–20 years (145). Since reoperation is a high-risk procedure for elderly patients and increases their mortality risk, in recent years, non-invasive implantation of a TAV inside the degenerative bioprosthetic valve has become an alternative intervention for these patients (145–147). However, it may come with the risk of coronary obstruction. Coronary obstruction is a rare consequence of TAVR that occurs during the procedure in <1% of patients, but it is life-threatening (128, 148, 149) as it restricts blood flow circulation in coronary arteries. Coronary obstruction can occur following a TAV implantation in native aortic valve or following a ViV procedure which includes TAV implantation inside another TAV or TAV implantation inside a surgical bioprosthetic valve. Coronary obstruction is more common during ViV procedure (about four times greater) than during TAVR in a native aortic valve (145, 146, 149), and more frequently occurs with use of balloon expandable valves (0.81% balloon expandable vs. 0.34% self-expandable) (149). This is possibly due to the differences in design and deployment mechanism of the transcatheter valves (148, 149). Coronary obstruction following a surgical bioprosthetic ViV procedure occurs more frequently in patients who had stentless or stented valves with bioprosthetic leaflets mounted externally (146). Additionally, it is proposed that coronary obstruction in surgical bioprosthetic ViV procedures is more related to the model and positioning of the surgical bioprosthetic valve, and is independent of the type of TAV, particularly if a surgical valve is implanted in a non-coaxial tilted position, decreasing the distance between leaflets and coronary ostia (148). Other surgical bioprosthetic valve risk factors were supra-annular implantation, high leaflet profile, valve design, stentless valves, or bulky bioprosthetic leaflets (148).

Clinical studies showed that anatomical factors such as low-laying coronary ostium and narrow sinus of Valsava (SOV), narrow sinotubular junction, and low sinus height are associated with coronary occlusion (148, 149), while the left coronary artery (LCA) more commonly becomes obstructed (88.6%) (149). In this study, the average height of LCA ostia in patients with coronary obstruction was 11 mm in men, and 10 mm in women. Most patients with SOV <30 mm and LCA ostium height <12 mm had coronary obstruction (146). Initially, female sex was identified as a risk factor for coronary obstruction (148, 149), but when aortic root dimensions were adjusted to body surface area, female anatomy was no longer an independent factor for coronary obstruction (148, 150).

Coronary obstruction can be caused by calcium deposits, a native leaflet blocking the coronary ostia, a TAV that is positioned too high within the annulus, or through thrombosis (128). In native TAVR procedures, coronary obstruction was linked to presence of bulky calcified lesions on the aortic leaflet blocking the coronary ostium (97.7%); however, the degree of calcification was not a predictor of coronary obstruction (149). Even though the location of the calcification is an important factor in coronary obstruction (148), to-date no study has been done to evaluate coronary obstruction with respect to anatomical features of coronary ostium and the location of the calcium nodules.

Coronary obstruction might be prevented by a novel intervention technique called bioprosthetic or native aortic scallop intentional laceration (BASILICA) (151).

## Bioprosthetic or Native Aortic Scallop Intentional Laceration of Coronary Artery

**Figure 4:**
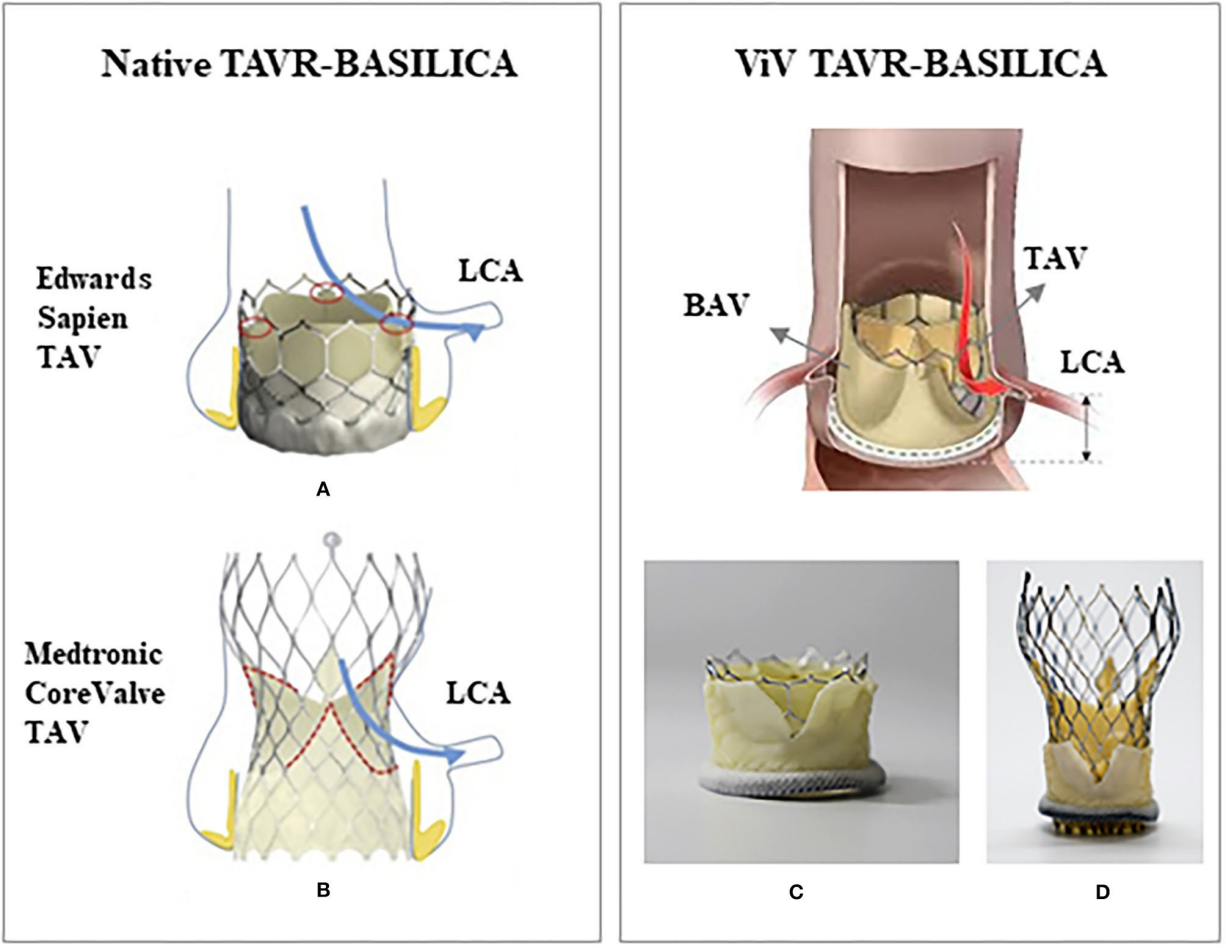
Schematic of a native TAVR-BASILICA and a valve-in-valve TAVR-BASILICA: **(A)** a Edwards Sapien 3 transcatheter aortic valve (TAV) and **(B)** a Medtronic CoreValve Evolut R TAV replaced in a native aortic valve. **(C)** An Edwards Sapien 3 TAV and **(D)** a Medtronic CoreValve Evolut R TAV replaced in a bioprosthetic aortic valve (BAV). Red and blue arrows show the location of the lacerated leaflet (native or bioprosthetic) relative to the left coronary artery (LCA). **(A,B)** are from Krishnaswamy et al. **(C,D)** are from Khodaee et al. with permission.

The first BASILICA human procedure was performed in 2011 during a surgical bioprosthetic ViV procedure in two patients using Edwards Sapien and Medtronic CoreValve to prevent coronary obstruction (147). This technique has been originated from the LAMPOON (Intentional Laceration of the Anterior Mitral leaflet to Prevent left ventricular Outflow Obstruction during transcatheter mitral valve implantation) technique (151). In this procedure, a guiding catheter carrying an electrified wire is directed toward aortic valve through the femoral artery and is positioned at the base of the leaflet; the electrified wire lacerates the leaflet from base to its free edge (152) and creates a split leaflet that would allow blood flow through the coronary arteries. Since the first BASILICA procedure in 2011, some clinical and computational studies have been performed to show the feasibility of BASILICA procedure and to evaluate its overall outcomes and outcomes relative to thrombosis and post-operation coronary obstruction (151–158); however implications of this procedure on outcomes remain unclear (Figure 4).

Leaflet thrombosis remains a concern for TAVR after the BASILICA procedure, despite theoretically creating more washout in the target aortic sinus and neosinus. A recent experimental study showed that leaflet laceration can mitigate the risk of thrombosis, while improving washout, with increases in velocity in the sinus and the neosinus by 50% for a Medtronic Evolve ViV, and more than 60% in Edwards Sapien 3 ViV (158). Similarly, a computational study showed that the average blood residence time (BRT) on the leaflets of BASILICA computational model was about 10% less than that in the ViV computational model without leaflet laceration. It has been hypothesized that thrombus is more likely to form in regions with low flow, which can better support fibrin formation due to low advective transport (or increased BRT) (157, 159, 160). Therefore, the BASILICA procedure appears to reduce the risk of leaflet thrombosis in the lacerated leaflets (157). Additionally, a computational study showed that the hemodynamic outcome of a two-leaflet-lacerated BASILICA model is improved when compared with a one-lacerated BASILICA model and the model without laceration, but no significant difference was observed for additional leaflet laceration (three-leaflet-lacerated model) (156). Overall, the BASILICA technique is still relatively new and require additional studies to better understand the benefits and when the procedure may be most effective.

## Discussion

As the general population has become older, the prevalence of calcific AS has increased in the recent decades; this has led to extensive research to reveal the complex underlying mechanisms of the valvular calcification, which involves mechanobiology, molecular signals, tissue remodeling, and inflammation, and yet our understanding of this complex process is limited.

Since pharmacotherapy has been ineffective in preventing progression of the calcification, treatment of calcific AS has become narrowed down to surgical and minimally-invasive interventions to repair or replace the native valve; this has led to design and development of artificial valves such as MHVs, BHVs, and TAVs that can mimic the function of the native valve. An immense amount of research has been performed to evaluate the performance of these artificial valves, and to develop better designs that can improve their flaws. Yet, there are undesirable post-interventional outcomes that are related to shortcomings of each valve design.

TAVR has gained favor as procedures and designs have undergone many improvements in recent decades. Despite this, there are still unresolved complications. New procedures aimed at overcoming challenges, like the BASILICA procedure continue to be investigated. Despite precise measurements on a patient's aortic valve anatomy and calcification, calcified lesions continue to complicate TAVR. Other tools like computational modeling have helped surgeons with pre-procedural planning, and with understanding the underlying biomechanics of post-procedural complications. However, these many of these tools continue to be validated. Overall, more studies are required to evaluate the relationships between new procedures and valves with hemodynamics, patient-specific anatomical characteristics, and deployment. This would help surgeons to select patients with suitable characteristics for specific procedures or valves that could improve outcomes.

## Author Contributions

BZ reviewed the literature and made figures. BZ and DB prepared the manuscript. All authors contributed to the article and approved the submitted version.

## Conflict of Interest

The authors declare that the research was conducted in the absence of any commercial or financial relationships that could be construed as a potential conflict of interest.

## Publisher's Note

All claims expressed in this article are solely those of the authors and do not necessarily represent those of their affiliated organizations, or those of the publisher, the editors and the reviewers. Any product that may be evaluated in this article, or claim that may be made by its manufacturer, is not guaranteed or endorsed by the publisher.

## References

[1] Dweck MR Boon NA Newby DE. Calcific aortic stenosis: a disease of the valve and the myocardium. J Am Coll Cardiol. (2012) 60:1854–63. 10.1016/j.jacc.2012.02.093 23062541

[2] Lindman BR Clavel M-A Mathieu P Iung B Lancellotti P Otto CM. Calcific aortic stenosis. Nat Rev Dis Primers. (2016) 2:1–28. 10.1038/nrdp.2016.6 27188578 PMC5127286

[3] Lindman BR Bonow RO Otto CM. Current management of calcific aortic stenosis. Circ Res. (2013) 113:223–37. 10.1161/CIRCRESAHA.111.300084 23833296 PMC4013234

[4] Otto CM. Calcific aortic stenosis-time to look more closely at the valve. N Engl J Med. (2008) 359:1395–8. 10.1056/NEJMe0807001 18815402

[5] Joseph J Naqvi SY Giri J Goldberg S. Aortic stenosis: pathophysiology, diagnosis, and therapy. Am J Med. (2017) 130:253–63. 10.1016/j.amjmed.2016.10.005 27810479

[6] Eveborn GW Schirmer H Heggelund G Lunde P Rasmussen K. The evolving epidemiology of valvular aortic stenosis. The Tromsø study. Heart. (2013) 99:396–400. 10.1136/heartjnl-2012-302265 22942293

[7] Sacks MS Smith DB Hiester ED. The aortic valve microstructure: effects of transvalvular pressure. J Biomed Mater Res. (1998) 41:131–41. 10.1002/(SICI)1097-4636(199807)41:1<131::AID-JBM16>3.0.CO;2-Q 9641633

[8] Gould ST Srigunapalan S Simmons CA Anseth KS. Hemodynamic and cellular response feedback in calcific aortic valve disease. Circ Res. (2013) 113:186–97. 10.1161/CIRCRESAHA.112.300154 23833293

[9] Stella JA Sacks MS. On the biaxial mechanical properties of the layers of the aortic valve leaflet. J Biomech Eng. (2007) 129:757–66. 10.1115/1.2768111 17887902

[10] Rutkovskiy A Malashicheva A Sullivan G Bogdanova M Kostareva A Stensløkken KO. Valve interstitial cells: the key to understanding the pathophysiology of heart valve calcification. J Am Heart Assoc. (2017) 6:e006339. 10.1161/JAHA.117.006339 28912209 PMC5634284

[11] Yip CYY Simmons CA. The aortic valve microenvironment and its role in calcific aortic valve disease. Cardiovasc Pathol. (2011) 20:177–82. 10.1016/j.carpath.2010.12.001 21256052

[12] Rajamannan NM Bonow RO Rahimtoola SH. Calcific aortic stenosis: an update. Nat Clin Pract Cardiovasc Med. (2007) 4:254–62. 10.1038/ncpcardio0827 17457349

[13] Rajamannan NM. Calcific aortic stenosis: lessons learned from experimental and clinical studies. Arterioscler Thromb Vasc Biol. (2009) 29:162–8. 10.1161/ATVBAHA.107.156752 19023094 PMC2774235

[14] Rajamannan NM Evans FJ Aikawa E Grande-Allen KJ Demer LL Heistad DD. Calcific aortic valve disease: not simply a degenerative process a review and agenda for research from the National Heart and Lung and Blood Institute Aortic Stenosis Working Group. Circulation. (2011) 124:1783. 10.1161/CIRCULATIONAHA.110.006767 22007101 PMC3306614

[15] Rajamannan NM Otto CM. Targeted therapy to prevent progression of calcific aortic stenosis. Circulation. (2004) 110:1180–2. 10.1161/01.CIR.0000140722.85490.EA 15353511 PMC3951855

[16] Lerman DA Prasad S Alotti N. Calcific aortic valve disease: molecular mechanisms and therapeutic approaches. Eur Cardiol Rev. (2015) 10:108. 10.15420/ecr.2015.10.2.108 27274771 PMC4888946

[17] Demer LL Tintut Y. Vascular calcification: pathobiology of a multifaceted disease. Circulation. (2008) 117:2938–48. 10.1161/CIRCULATIONAHA.107.743161 18519861 PMC4431628

[18] Mohler ER Nichols R Harvey W Sheridan M Waller B Waller BF. Development and progression of aortic valve stenosis: atherosclerosis risk factors-a causal relationship? A clinical morphologic study. Clin Cardiol. (1991) 14:995–9. 10.1002/clc.4960141210 1841025

[19] Butcher JT Nerem RM. Valvular endothelial cells and the mechanoregulation of valvular pathology. Philos Trans R Soc B Biol Sci. (2007) 362:1445–57. 10.1098/rstb.2007.2127 17569641 PMC2440407

[20] Fernández Esmerats J Heath J Jo H. Shear-sensitive genes in aortic valve endothelium. Antioxid Redox Signal. (2016) 25:401–14. 10.1089/ars.2015.6554 26651130 PMC5011632

[21] Tarbell JM Shi Z-D Dunn J Jo H. Fluid mechanics, arterial disease, and gene expression. Annu Rev Fluid Mech. (2014) 46:591–614. 10.1146/annurev-fluid-010313-141309 25360054 PMC4211638

[22] Katritch V Cherezov V Stevens RC. Structure-function of the G protein-coupled receptor superfamily. Annu Rev Pharmacol Toxicol. (2013) 53:531–56. 10.1146/annurev-pharmtox-032112-135923 23140243 PMC3540149

[23] Lombardi ML Jaalouk DE Shanahan CM Burke B Roux KJ Lammerding J. The interaction between nesprins and sun proteins at the nuclear envelope is critical for force transmission between the nucleus and cytoskeleton. J Biol Chem. (2011) 286:26743–53. 10.1074/jbc.M111.233700 21652697 PMC3143636

[24] Aikawa E Nahrendorf M Figueiredo J-L Swirski FK Shtatland T Kohler RH. CLINICAL PERSPECTIVE. Circulation. (2007) 116:2841–50. 10.1161/CIRCULATIONAHA.107.732867 18040026

[25] Hjortnaes J New SE Aikawa E. Visualizing novel concepts of cardiovascular calcification. Trends Cardiovasc Med. (2013) 23:71–9. 10.1016/j.tcm.2012.09.003 23290463 PMC3626075

[26] Nigam V Srivastava D. Notch1 represses osteogenic pathways in aortic valve cells. J Mol Cell Cardiol. (2009) 47:828–34. 10.1016/j.yjmcc.2009.08.008 19695258 PMC2783189

[27] Yutzey KE Demer LL Body SC Huggins GS Towler DA Giachelli CM. Calcific aortic valve disease: a consensus summary from the Alliance of Investigators on Calcific Aortic Valve Disease. Arterioscler Thromb Vasc Biol. (2014) 34:2387–93. 10.1161/ATVBAHA.114.302523 25189570 PMC4199903

[28] Kawakami R Nakagami H Noma T Ohmori K Kohno M Morishita R. RANKL system in vascular and valve calcification with aging. Inflamm Regen. (2016) 36:1–6. 10.1186/s41232-016-0016-3 29259683 PMC5725909

[29] Kaden JJ Bickelhaupt S Grobholz R Haase KK Sarιkoç A Brueckmann M. Receptor activator of nuclear factor κB ligand and osteoprotegerin regulate aortic valve calcification. J Mol Cell Cardiol. (2004) 36:57–66. 10.1016/j.yjmcc.2003.09.015 14734048

[30] Rajamannan NM Subramaniam M Caira F Stock SR Spelsberg TC. Atorvastatin inhibits hypercholesterolemia-induced calcification in the aortic valves via the Lrp5 receptor pathway. Circulation. (2005) 112:I-229–I-34. 10.1161/01.CIRCULATIONAHA.104.524306 16159822 PMC3951868

[31] O'Brien KD. Pathogenesis of calcific aortic valve disease: a disease process comes of age (and a good deal more). Arterioscler Thromb Vasc Biol. (2006) 26:1721–8. 10.1161/01.ATV.0000227513.13697.ac 16709942

[32] Phimphilai M Zhao Z Boules H Roca H Franceschi RT. BMP signaling is required for RUNX2-dependent induction of the osteoblast phenotype. J Bone Miner Res. (2006) 21:637–46. 10.1359/jbmr.060109 16598384 PMC2435171

[33] Favero G Paganelli C Buffoli B Rodella LF Rezzani R. Endothelium and its alterations in cardiovascular diseases: life style intervention. Biomed Res Int. (2014) 2014:801896. 10.1155/2014/801896 24719887 PMC3955677

[34] Thanassoulis G. Lipoprotein (a) in calcific aortic valve disease: from genomics to novel drug target for aortic stenosis. J Lipid Res. (2016) 57:917–24. 10.1194/jlr.R051870 26685327 PMC4878194

[35] Cairns BJ Coffey S Travis RC Prendergast B Green J Engert JC. A replicated, genome-wide significant association of aortic stenosis with a genetic variant for lipoprotein (a) meta-analysis of published and novel data. Circulation. (2017) 135:1181–3. 10.1161/CIRCULATIONAHA.116.026103 28320808

[36] Hoekstra M Chen HY Rong J Dufresne L Yao J Guo X. Genome-wide association study highlights APOH as a novel locus for lipoprotein (a) levels-brief report. Arterioscler Thromb Vasc Biol. (2021) 41:458–64. 10.1161/ATVBAHA.120.314965 33115273 PMC7769958

[37] Passos LS Lupieri A Becker-Greene D Aikawa E. Innate and adaptive immunity in cardiovascular calcification. Atherosclerosis. (2020) 306:59–67. 10.1016/j.atherosclerosis.2020.02.016 32222287 PMC7483874

[38] Akin I Nienaber CA. Is there evidence for statins in the treatment of aortic valve stenosis? World J Cardiol. (2017) 9:667. 10.4330/wjc.v9.i8.667 28932355 PMC5583539

[39] Cowell SJ Newby DE Prescott RJ Bloomfield P Reid J Northridge DB. A randomized trial of intensive lipid-lowering therapy in calcific aortic stenosis. N Engl J Med. (2005) 352:2389–97. 10.1056/NEJMoa043876 15944423

[40] Chan KL Teo K Dumesnil JG Ni A Tam J. Effect of Lipid lowering with rosuvastatin on progression of aortic stenosis: results of the aortic stenosis progression observation: measuring effects of rosuvastatin (ASTRONOMER) trial. Circulation. (2010) 121:306–14. 10.1161/CIRCULATIONAHA.109.900027 20048204

[41] Rossebø AB Pedersen TR Boman K Brudi P Chambers JB Egstrup K. Intensive lipid lowering with simvastatin and ezetimibe in aortic stenosis. N Engl J Med. (2008) 359:1343–56. 10.1056/NEJMoa0804602 18765433

[42] Freeman RV Otto CM. Spectrum of calcific aortic valve disease: pathogenesis, disease progression, and treatment strategies. Circulation. (2005) 111:3316–26. 10.1161/CIRCULATIONAHA.104.486738 15967862

[43] Santibáñez JF Guerrero J Quintanilla M Fabra A Martínez J. Transforming growth factor-β1 modulates matrix metalloproteinase-9 production through the Ras/MAPK signaling pathway in transformed keratinocytes. Biochem Biophys Res Commun. (2002) 296:267–73. 10.1016/S0006-291X(02)00864-1 12163012

[44] Helderman F Segers D de Crom R Hierck BP Poelmann RE Evans PC. Effect of shear stress on vascular inflammation and plaque development. Curr Opin Lipidol. (2007) 18:527–33. 10.1097/MOL.0b013e3282ef7716 17885423

[45] Kaden JJ Dempfle C-E Grobholz R Fischer CS Vocke DC Kiliç R. Inflammatory regulation of extracellular matrix remodeling in calcific aortic valve stenosis. Cardiovasc Pathol. (2005) 14:80–7. 10.1016/j.carpath.2005.01.002 15780799

[46] Mathieu P Bouchareb R Boulanger M-C. Innate and adaptive immunity in calcific aortic valve disease. J Immunol Res. (2015) 2015:851945. 10.1155/2015/851945 26065007 PMC4433691

[47] New SE Goettsch C Aikawa M Marchini JF Shibasaki M Yabusaki K. Macrophage-derived matrix vesicles: an alternative novel mechanism for microcalcification in atherosclerotic plaques. Circ Res. (2013) 113:72–7. 10.1161/CIRCRESAHA.113.301036 23616621 PMC3703850

[48] Kapustin AN Chatrou ML Drozdov I Zheng Y Davidson SM Soong D. Vascular smooth muscle cell calcification is mediated by regulated exosome secretion. Circ Res. (2015) 116:1312–23. 10.1161/CIRCRESAHA.116.305012 25711438

[49] Myasoedova VA Ravani AL Frigerio B Valerio V Moschetta D Songia P. Novel pharmacological targets for calcific aortic valve disease: prevention and treatments. Pharmacol Res. (2018) 136:74–82. 10.1016/j.phrs.2018.08.020 30149054

[50] Graham MJ Viney N Crooke RM Tsimikas S. Antisense inhibition of apolipoprotein (a) to lower plasma lipoprotein (a) levels in humans. J Lipid Res. (2016) 57:340–51. 10.1194/jlr.R052258 26538546 PMC4767000

[51] Tsimikas S Viney NJ Hughes SG Singleton W Graham MJ Baker BF. Antisense therapy targeting apolipoprotein (a): a randomised, double-blind, placebo-controlled phase 1 study. Lancet. (2015) 386:1472–83. 10.1016/S0140-6736(15)61252-1 26210642

[52] Stein EA Raal F. Future directions to establish lipoprotein (a) as a treatment for atherosclerotic cardiovascular disease. Cardiovasc Drugs Therapy. (2016) 30:101–8. 10.1007/s10557-016-6654-5 26861250

[53] Garg A Sharma A Krishnamoorthy P Garg J Virmani D Sharma T. Role of niacin in current clinical practice: a systematic review. Am J Med. (2017) 130:173–87. 10.1016/j.amjmed.2016.07.038 27793642

[54] Albers JJ Slee A O'Brien KD Robinson JG Kashyap ML Kwiterovich PO. Relationship of apolipoproteins A-1 and B, and lipoprotein (a) to cardiovascular outcomes: the AIM-HIGH trial (Atherothrombosis Intervention in Metabolic Syndrome with Low HDL/High Triglyceride and Impact on Global Health Outcomes). J Am Coll Cardiol. (2013) 62:1575–9. 10.1016/j.jacc.2013.06.051 23973688 PMC3800510

[55] Raal FJ Giugliano RP Sabatine MS Koren MJ Blom D Seidah NG. PCSK9 inhibition-mediated reduction in Lp (a) with evolocumab: an analysis of 10 clinical trials and the LDL receptor's role [S]. J Lipid Res. (2016) 57:1086–96. 10.1194/jlr.P065334 27102113 PMC4878192

[56] Sabatine MS Giugliano RP Keech AC Honarpour N Wiviott SD Murphy SA. Evolocumab and clinical outcomes in patients with cardiovascular disease. N Engl J Med. (2017) 376:1713–22. 10.1056/NEJMoa1615664 28304224

[57] Seya K Furukawa K-I Chiyoya M Yu Z Kikuchi H Daitoku K. 1-Methyl-2-undecyl-4 (1H)-quinolone, a derivative of quinolone alkaloid evocarpine, attenuates high phosphate-induced calcification of human aortic valve interstitial cells by inhibiting phosphate cotransporter PiT-1. J Pharmacol Sci. (2016) 131:51–7. 10.1016/j.jphs.2016.04.013 27165707

[58] Miller JD Weiss RM Serrano KM Brooks RM Berry CJ Zimmerman K. Lowering plasma cholesterol levels halts progression of aortic valve disease in mice. Circulation. (2009) 119:2693–701. 10.1161/CIRCULATIONAHA.108.834614 19433756 PMC2740986

[59] Bouchareb R Côté N Le Quang K El Husseini D Asselin J Hadji F. Carbonic anhydrase XII in valve interstitial cells promotes the regression of calcific aortic valve stenosis. J Mol Cell Cardiol. (2015) 82:104–15. 10.1016/j.yjmcc.2015.03.002 25771146

[60] Carabello BA. How does the heart respond to aortic stenosis: let me count the ways. Am Heart Assoc. (2013) 6:858–60. 10.1161/CIRCIMAGING.113.001242 24254476

[61] Cowell SJ Newby DE Boon NA Elder AT. Calcific aortic stenosis: same old story? Age Ageing. (2004) 33:538–44. 10.1093/ageing/afh175 15308457

[62] Saikrishnan N Kumar G Sawaya FJ Lerakis S Yoganathan AP. Accurate assessment of aortic stenosis: a review of diagnostic modalities and hemodynamics. Circulation. (2014) 129:244–53. 10.1161/CIRCULATIONAHA.113.002310 24421359

[63] Kwon S Gopal A. Hemodynamic classifications of aortic stenosis and relevance to prognosis. Aortic Stenosis Curr Perspect. (2019) 1–18. 10.5772/intechopen.86707

[64] Baumgartner H Hung J Bermejo J Chambers JB Evangelista A Griffin BP. Echocardiographic assessment of valve stenosis: EAE/ASE recommendations for clinical practice. J Am Soc Echocardiogr. (2009) 22:1–23. 10.1016/j.echo.2008.11.029 19130998

[65] Garcia J Kadem L Larose E Clavel M-A Pibarot P. Comparison between cardiovascular magnetic resonance and transthoracic Doppler echocardiography for the estimation of effective orifice area in aortic stenosis. J Cardiovasc Magn Resonance. (2011) 13:1–9. 10.1186/1532-429X-13-25 21527021 PMC3108925

[66] Agatston AS Janowitz WR Hildner FJ Zusmer NR Viamonte M Detrano R. Quantification of coronary artery calcium using ultrafast computed tomography. J Am Coll Cardiol. (1990) 15:827–32. 10.1016/0735-1097(90)90282-T 2407762

[67] Koos R Mahnken AH Sinha AM Wildberger JE Hoffmann R Kühl HP. Aortic valve calcification as a marker for aortic stenosis severity: assessment on 16-MDCT. Am J Roentgenol. (2004) 183:1813–8. 10.2214/ajr.183.6.01831813 15547235

[68] Pawade T Clavel M-A Tribouilloy C Dreyfus J Mathieu T Tastet L. Computed tomography aortic valve calcium scoring in patients with aortic stenosis. Circulation. (2018) 11:e007146. 10.1161/CIRCIMAGING.117.007146 29555836

[69] Clavel M-A Messika-Zeitoun D Pibarot P Aggarwal SR Malouf J Araoz PA. The complex nature of discordant severe calcified aortic valve disease grading: new insights from combined Doppler echocardiographic and computed tomographic study. J Am Coll Cardiol. (2013) 62:2329–38. 10.1016/j.jacc.2013.08.1621 24076528

[70] Clavel M-A Pibarot P Messika-Zeitoun D Capoulade R Malouf J Aggarwal SR. Impact of aortic valve calcification, as measured by MDCT, on survival in patients with aortic stenosis: results of an international registry study. J Am Coll Cardiol. (2014) 64:1202–13. 10.1016/j.jacc.2014.05.066 25236511 PMC4391203

[71] Voisine M Hervault M Shen M Boilard AJ Filion B Rosa M. Age, sex, and valve phenotype differences in fibro-calcific remodeling of calcified aortic valve. J Am Heart Assoc. (2020) 9:e015610. 10.1161/JAHA.119.015610 32384012 PMC7660864

[72] Summerhill VI Moschetta D Orekhov AN Poggio P Myasoedova VA. Sex-specific features of calcific aortic valve disease. Int J Mol Sci. (2020) 21:5620. 10.3390/ijms21165620 32781508 PMC7460640

[73] Fleury M-A Clavel M-A. Sex and race differences in the pathophysiology, diagnosis, treatment, and outcomes of valvular heart diseases. Can J Cardiol. (2021) 37:980–91. 10.1016/j.cjca.2021.02.003 33581193

[74] Osako MK Nakagami H Koibuchi N Shimizu H Nakagami F Koriyama H. Estrogen inhibits vascular calcification via vascular RANKL system: common mechanism of osteoporosis and vascular calcification. Circ Res. (2010) 107:466–75. 10.1161/CIRCRESAHA.110.216846 20595654

[75] Harper E Forde H Davenport C Rochfort KD Smith D Cummins PM. Vascular calcification in type-2 diabetes and cardiovascular disease: Integrative roles for OPG, RANKL and TRAIL. Vascul Pharmacol. (2016) 82:30–40. 10.1016/j.vph.2016.02.003 26924459

[76] Zhang B Miller VM Miller JD. Influences of sex and estrogen in arterial and valvular calcification. Front Endocrinol. (2019) 10:622. 10.3389/fendo.2019.00622 31620082 PMC6763561

[77] Gelfand ML Cohen T Ackert JJ Ambos M Mayadag M. Gastrointestinal bleeding in aortic stenosis. Am J Gastroenterol. (1979) 71:30–8. 312009

[78] Warkentin TE Moore JC Morgan DG. Gastrointestinal angiodysplasia and aortic stenosis. N Engl J Med. (2002) 347:858–9. 10.1056/NEJM200209123471122 12226167

[79] Vincentelli A Susen S Le Tourneau T Six I Fabre O Juthier F. Acquired von Willebrand syndrome in aortic stenosis. N Engl J Med. (2003) 349:343–9. 10.1056/NEJMoa022831 12878741

[80] Nkomo VT Gardin JM Skelton TN Gottdiener JS Scott CG Enriquez-Sarano M. Burden of valvular heart diseases: a population-based study. Lancet. (2006) 368:1005–11. 10.1016/S0140-6736(06)69208-8 16980116

[81] Yasar SJ Abdullah O Fay W Balla S. Von Willebrand factor revisited. J Interv Cardiol. (2018) 31:360–7. 10.1111/joic.12478 29285810

[82] Susen S Vincentelli A Le Tourneau T Caron C Zawadzki C Prat A. Severe aortic and mitral valve regurgitation are associated with von willebrand factor defect. Am Soc Hematol. (2005) 106:1790. 10.1182/blood.V106.11.1790.179016095168

[83] Blackshear J Wysokinska E Safford R Thomas C Shapiro B Ung S. Shear stress-associated acquired Von Willebrand syndrome in patients with mitral regurgitation. J Thromb Haemost. (2014) 12:1966–74. 10.1111/jth.12734 25251907

[84] Van Belle E Rauch A Vincent F Robin E Kibler M Labreuche J. Von Willebrand factor multimers during transcatheter aortic-valve replacement. N Engl J Med. (2016) 375:335–44. 10.1056/NEJMoa1505643 27464202

[85] Van Belle E Vincent F Rauch A Casari C Jeanpierre E Loobuyck V. von Willebrand factor and management of heart valve disease: JACC review topic of the week. J Am Coll Cardiol. (2019) 73:1078–88. 10.1016/j.jacc.2018.12.045 30846101

[86] Bortot M Bark K Neeves K Clendenen N Bark DJ DiPaola J. Impaired primary hemostasis in patients on cardiopulmonary bypass. Arterioscler Thromb Vasc Biol. (2019) 39:A130. 10.1161/atvb.39.suppl_1.130

[87] Cohn LH Tchantchaleishvili V Rajab TK. Evolution of the concept and practice of mitral valve repair. Ann Cardiothorac Surg. (2015) 4:315–21. 10.3978/j.issn.2225-319X.2015.04.09 26309840 PMC4526492

[88] Aicher D Fries R Rodionycheva S Schmidt K Langer F Schäfers H-J. Aortic valve repair leads to a low incidence of valve-related complications. Eur J Cardio Thorac Surg. (2010) 37:127–32. 10.1016/j.ejcts.2009.06.021 19643618

[89] Cribier A Saoudi N Berland J Savin T Rocha P Letac B. Percutaneous transluminal valvuloplasty of acquired aortic stenosis in elderly patients: an alternative to valve replacement? Lancet. (1986) 327:63–7. 10.1016/S0140-6736(86)90716-6 2867315

[90] Safian RD Mandell VS Thurer RE Hutchins GM Schnitt SJ Grossman W. Postmortem and intraoperative balloon valvuloplasty of calcific aortic stenosis in elderly patients: mechanisms of successful dilation. J Am Coll Cardiol. (1987) 9:655–60. 10.1016/S0735-1097(87)80061-X 2950156

[91] Arzt W Wertaschnigg D Veit I Klement F Gitter R Tulzer G. Intrauterine aortic valvuloplasty in fetuses with critical aortic stenosis: experience and results of 24 procedures. Ultrasound Obstetr Gynecol. (2011) 37:689–95. 10.1002/uog.8927 21229549

[92] Marshall AC Tworetzky W Bergersen L McElhinney DB Benson CB Jennings RW. Aortic valvuloplasty in the fetus: technical characteristics of successful balloon dilation. J Pediatr. (2005) 147:535–9. 10.1016/j.jpeds.2005.04.055 16227042

[93] Tworetzky W Wilkins-Haug L Jennings RW van der Velde ME Marshall AC Marx GR. Balloon dilation of severe aortic stenosis in the fetus: potential for prevention of hypoplastic left heart syndrome: candidate selection, technique, and results of successful intervention. Circulation. (2004) 110:2125–31. 10.1161/01.CIR.0000144357.29279.54 15466631

[94] Friedman KG Margossian R Graham DA Harrild DM Emani SM Wilkins-Haug LE. Postnatal left ventricular diastolic function after fetal aortic valvuloplasty. Am J Cardiol. (2011) 108:556–60. 10.1016/j.amjcard.2011.03.085 21624551 PMC3374950

[95] Kogoj P Devjak R Bunc M. Balloon aortic valvuloplasty (BAV) as a bridge to aortic valve replacement in cancer patients who require urgent non-cardiac surgery. Radiol Oncol. (2014) 48:62. 10.2478/raon-2013-0078 24587781 PMC3908849

[96] Head SJ Çelik M Kappetein AP. Mechanical versus bioprosthetic aortic valve replacement. Eur Heart J. (2017) 38:2183–91. 10.1093/eurheartj/ehx141 28444168

[97] Pibarot P Salaun E Dahou A Avenatti E Guzzetti E Annabi M-S. Echocardiographic results of transcatheter versus surgical aortic valve replacement in low-risk patients: the PARTNER 3 trial. Circulation. (2020) 141:1527–37. 10.1161/CIRCULATIONAHA.119.044574 32272848

[98] Braghiroli J Kapoor K Thielhelm TP Ferreira T Cohen MG. Transcatheter aortic valve replacement in low risk patients: a review of PARTNER 3 and Evolut low risk trials. Cardiovasc Diagn Ther. (2020) 10:59. 10.21037/cdt.2019.09.12 32175228 PMC7044101

[99] Cribier A Eltchaninoff H Bash A Borenstein N Tron C Bauer F. Percutaneous transcatheter implantation of an aortic valve prosthesis for calcific aortic stenosis: first human case description. Circulation. (2002) 106:3006–8. 10.1161/01.CIR.0000047200.36165.B8 12473543

[100] Kheradvar A Groves EM Goergen CJ Alavi SH Tranquillo R Simmons CA. Emerging trends in heart valve engineering: Part II. Novel and standard technologies for aortic valve replacement. Ann Biomed Eng. (2015) 43:844–57. 10.1007/s10439-014-1191-5 25449148

[101] Lancellotti P Vannan MA. Timing of intervention in aortic stenosis. N Engl J Med. (2020) 382:191–3. 10.1056/NEJMe1914382 31733179

[102] Banovic M Iung B Putnik S Nikolic S Penicka M Deja M. Addressing the treatment dilemma in asymptomatic aortic stenosis: the AVATAR trial. JACC Cardiovasc Imaging. (2019) 12:1896–7. 10.1016/j.jcmg.2019.07.012 31488255

[103] James Everett R Clavel M-A Pibarot P Dweck MR. Timing of intervention in aortic stenosis a review of current and future strategies. Heart. (2018) 104:2067–76. 10.1136/heartjnl-2017-312304 30030337 PMC6287563

[104] Lindman BR Dweck MR Lancellotti P Généreux P Piérard LA O'Gara PT. Management of asymptomatic severe aortic stenosis: evolving concepts in timing of valve replacement. Cardiovasc Imaging. (2020) 13:481–93. 10.1016/j.jcmg.2019.01.036 31202751

[105] Smith CR Leon MB Mack MJ Miller DC Moses JW Svensson LG. Transcatheter versus surgical aortic-valve replacement in high-risk patients. N Engl J Med. (2011) 364:2187–98. 10.1056/NEJMoa1103510 21639811

[106] Gleason TG Reardon MJ Popma JJ Deeb GM Yakubov SJ Lee JS. 5-Year outcomes of self-expanding transcatheter versus surgical aortic valve replacement in high-risk patients. J Am Coll Cardiol. (2018) 72:2687–96. 10.1016/j.jacc.2018.08.2146 30249462

[107] Mack MJ Leon MB Smith CR Miller DC Moses JW Tuzcu EM. 5-year outcomes of transcatheter aortic valve replacement or surgical aortic valve replacement for high surgical risk patients with aortic stenosis (PARTNER 1): a randomised controlled trial. Lancet. (2015) 385:2477–84. 10.1016/S0140-6736(15)60308-7 25788234

[108] Kolte D Vlahakes GJ Palacios IF Sakhuja R Passeri JJ Inglessis I. Transcatheter versus surgical aortic valve replacement in low-risk patients. J Am Coll Cardiol. (2019) 74:1532–40. 10.1016/j.jacc.2019.06.076 31537261

[109] Grines CL Klein AJ Bauser-Heaton H Alkhouli M Katukuri N Aggarwal V. Racial and ethnic disparities in coronary, vascular, structural, and congenital heart disease. Catheter Cardiovasc Interv. (2021) 98:277–94. 10.1002/ccd.29745 33909339

[110] Alkhouli M Holmes DR Carroll JD Li Z Inohara T Kosinski AS. Racial disparities in the utilization and outcomes of TAVR: TVT registry report. JACC Cardiovasc Interv. (2019) 12:936–48. 10.1016/j.jcin.2019.03.007 31122351

[111] Sleder A Tackett S Cerasale M Mittal C Isseh I Radjef R. Socioeconomic and racial disparities: a case-control study of patients receiving transcatheter aortic valve replacement for severe aortic stenosis. J Racial Ethnic Health Disparities. (2017) 4:1189–94. 10.1007/s40615-016-0325-x 28039604

[112] Yeung M Kerrigan J Sodhi S Huang P-H Novak E Maniar H. Racial differences in rates of aortic valve replacement in patients with severe aortic stenosis. Am J Cardiol. (2013) 112:991–5. 10.1016/j.amjcard.2013.05.030 23791013

[113] Henn MC Moon MR. Mechanical prosthetic valves. In: Raja S editor. Cardiac Surgery. Cham: Springer (2020). p. 291–8.

[114] Alemu Y Bluestein D. Flow-induced platelet activation and damage accumulation in a mechanical heart valve: numerical studies. Artif Organs. (2007) 31:677–88. 10.1111/j.1525-1594.2007.00446.x 17725695

[115] Wootton DM Ku DN. Fluid mechanics of vascular systems, diseases, and thrombosis. Annu Rev Biomed Eng. (1999) 1:299–329. 10.1146/annurev.bioeng.1.1.299 11701491

[116] Yun BM Wu J Simon HA Arjunon S Sotiropoulos F Aidun CK. A numerical investigation of blood damage in the hinge area of aortic bileaflet mechanical heart valves during the leakage phase. Ann Biomed Eng. (2012) 40:1468–85. 10.1007/s10439-011-0502-3 22215278

[117] Ge L Dasi LP Sotiropoulos F Yoganathan AP. Characterization of hemodynamic forces induced by mechanical heart valves: Reynolds vs. viscous stresses. Ann Biomed Eng. (2008) 36:276–97. 10.1007/s10439-007-9411-x 18049902

[118] Dangas GD Weitz JI Giustino G Makkar R Mehran R. Prosthetic heart valve thrombosis. J Am Coll Cardiol. (2016) 68:2670–89. 10.1016/j.jacc.2016.09.958 27978952

[119] Kulik A Rubens FD Wells PS Kearon C Mesana TG van Berkom J. Early postoperative anticoagulation after mechanical valve replacement: a systematic review. Ann Thorac Surg. (2006) 81:770–81. 10.1016/j.athoracsur.2005.07.023 16427905

[120] Sun T Tan H Han D Fu Q Jiang L. No platelet can adhere-largely improved blood compatibility on nanostructured superhydrophobic surfaces. Small. (2005) 1:959–63. 10.1002/smll.200500095 17193377

[121] Bark DL Vahabi H Bui H Movafaghi S Moore B Kota AK. Hemodynamic performance and thrombogenic properties of a superhydrophobic bileaflet mechanical heart valve. Ann Biomed Eng. (2017) 45:452–63. 10.1007/s10439-016-1618-2 27098219 PMC5073049

[122] Leslie DC Waterhouse A Berthet JB Valentin TM Watters AL Jain A. A bioinspired omniphobic surface coating on medical devices prevents thrombosis and biofouling. Nat Biotechnol. (2014) 32:1134–40. 10.1038/nbt.3020 25306244

[123] Khorasani M Mirzadeh H. In vitro blood compatibility of modified PDMS surfaces as superhydrophobic and superhydrophilic materials. J Appl Polym Sci. (2004) 91:2042–7. 10.1002/app.13355

[124] Johnston DR Soltesz EG Vakil N Rajeswaran J Roselli EE Sabik JF III. Long-term durability of bioprosthetic aortic valves: implications from 12,569 implants. Ann Thorac Surg. (2015) 99:1239–47. 10.1016/j.athoracsur.2014.10.070 25662439 PMC5132179

[125] Dasi LP Hatoum H Kheradvar A Zareian R Alavi SH Sun W. On the mechanics of transcatheter aortic valve replacement. Ann Biomed Eng. (2017) 45:310–31. 10.1007/s10439-016-1759-3 27873034 PMC5300937

[126] Alavi SH Groves EM Kheradvar A. The effects of transcatheter valve crimping on pericardial leaflets. Ann Thorac Surg. (2014) 97:1260–6. 10.1016/j.athoracsur.2013.11.009 24444873

[127] Del Pino MDCL Ortiz MR Ortega MD Fernández JS Quero CF Jiménez ED. Prosthesis-patient mismatch after transcatheter aortic valve replacement: prevalence and medium term prognostic impact. Int J Cardiovasc Imaging. (2019) 35:827–36. 10.1007/s10554-018-01519-z 30661140

[128] Laborde J-C Brecker SJ Roy D Jahangiri M. Complications at the time of transcatheter aortic valve implantation. Methodist Debakey Cardiovasc J. (2012) 8:38. 10.14797/mdcj-8-2-38 22891127 PMC3405794

[129] Makkar RR Jilaihawi H Chakravarty T Fontana GP Kapadia S Babaliaros V. Determinants and outcomes of acute transcatheter valve-in-valve therapy or embolization: a study of multiple valve implants in the US PARTNER trial (Placement of AoRTic TraNscathetER Valve Trial Edwards SAPIEN Transcatheter Heart Valve). J Am Coll Cardiol. (2013) 62:418–30. 10.1016/j.jacc.2013.04.037 23684680

[130] Fassa A-A Himbert D Vahanian A. Mechanisms and management of TAVR-related complications. Nat Rev Cardiol. (2013) 10:685. 10.1038/nrcardio.2013.156 24101101

[131] Barbanti M Yang T-H Rodès Cabau J Tamburino C Wood DA Jilaihawi H. Anatomical and procedural features associated with aortic root rupture during balloon-expandable transcatheter aortic valve replacement. Circulation. (2013) 128:244–53. 10.1161/CIRCULATIONAHA.113.002947 23748467

[132] Blanke P Reinöhl J Schlensak C Siepe M Pache G Euringer W. Prosthesis oversizing in balloon-expandable transcatheter aortic valve implantation is associated with contained rupture of the aortic root. Circulation. (2012) 5:540–8. 10.1161/CIRCINTERVENTIONS.111.967349 22872051

[133] Willson AB Webb JG Freeman M Wood DA Gurvitch R Thompson CR. Computed tomography-based sizing recommendations for transcatheter aortic valve replacement with balloon-expandable valves: comparison with transesophageal echocardiography and rationale for implementation in a prospective trial. J Cardiovasc Comput Tomogr. (2012) 6:406–14. 10.1016/j.jcct.2012.10.002 23127390

[134] Binder RK Webb JG Willson AB Urena M Hansson NC Norgaard BL. The impact of integration of a multidetector computed tomography annulus area sizing algorithm on outcomes of transcatheter aortic valve replacement: a prospective, multicenter, controlled trial. J Am Coll Cardiol. (2013) 62:431–8. 10.1016/j.jacc.2013.04.036 23684679

[135] Bernard S Yucel E. Paravalvular leaks-from diagnosis to management. Curr Treat Options Cardiovasc Med. (2019) 21:1–16. 10.1007/s11936-019-0776-6 31728667

[136] Gilard M Eltchaninoff H Iung B Donzeau-Gouge P Chevreul K Fajadet J. Registry of transcatheter aortic-valve implantation in high-risk patients. N Engl J Med. (2012) 366:1705–15. 10.1056/NEJMoa1114705 22551129

[137] Moat NE Ludman P de Belder MA Bridgewater B Cunningham AD Young CP. Long-term outcomes after transcatheter aortic valve implantation in high-risk patients with severe aortic stenosis: the UK TAVI (United Kingdom Transcatheter Aortic Valve Implantation) Registry. J Am Coll Cardiol. (2011) 58:2130–8. 10.1016/j.jacc.2011.08.050 22019110

[138] Athappan G Patvardhan E Tuzcu EM Svensson LG Lemos PA Fraccaro C. Incidence, predictors, and outcomes of aortic regurgitation after transcatheter aortic valve replacement: meta-analysis and systematic review of literature. J Am Coll Cardiol. (2013) 61:1585–95. 10.1016/j.jacc.2013.01.047 23500308

[139] Gilbert ON Choi CH Franzil JL Caughey M Qureshi W Stacey RB. Comparison of paravalvular aortic leak characteristics in the Medtronic CoreValve versus Edwards Sapien Valve: paravalvular aortic leak characteristics. Catheter Cardiovasc Interv. (2018) 92:972–80. 10.1002/ccd.27643 29726601

[140] Franzoni I Latib A Maisano F Costopoulos C Testa L Figini F. Comparison of incidence and predictors of left bundle branch block after transcatheter aortic valve implantation using the CoreValve versus the Edwards valve. Am J Cardiol. (2013) 112:554–9. 10.1016/j.amjcard.2013.04.026 23726173

[141] Siontis GC Jüni P Pilgrim T Stortecky S Büllesfeld L Meier B. Predictors of permanent pacemaker implantation in patients with severe aortic stenosis undergoing TAVR: a meta-analysis. J Am Coll Cardiol. (2014) 64:129–40. 10.1016/j.jacc.2014.04.033 25011716

[142] Khatri PJ Webb JG Rodés-Cabau J Fremes SE Ruel M Lau K. Adverse effects associated with transcatheter aortic valve implantation: a meta-analysis of contemporary studies. Ann Intern Med. (2013) 158:35–46. 10.7326/0003-4819-158-1-201301010-00007 23277899

[143] Karyofillis P Kostopoulou A Thomopoulou S Habibi M Livanis E Karavolias G. Conduction abnormalities after transcatheter aortic valve implantation. J Geriatr Cardiol. (2018) 15:105–12. 10.11909/j.issn.1671-5411.2018.01.004 29434632 PMC5803544

[144] Khawaja M Rajani R Cook A Khavandi A Moynagh A Chowdhary S. Permanent pacemaker insertion after CoreValve transcatheter aortic valve implantation: incidence and contributing factors (the UK CoreValve Collaborative). Circulation. (2011) 123:951–60. 10.1161/CIRCULATIONAHA.109.927152 21339482

[145] Dvir D Webb J Brecker S Bleiziffer S Hildick-Smith D Colombo A. Transcatheter aortic valve replacement for degenerative bioprosthetic surgical valves: results from the global valve-in-valve registry. Circulation. (2012) 126:2335–44. 10.1161/CIRCULATIONAHA.112.104505 23052028

[146] Ribeiro HB Rodés-Cabau J Blanke P Leipsic J Kwan Park J Bapat V. Incidence, predictors, and clinical outcomes of coronary obstruction following transcatheter aortic valve replacement for degenerative bioprosthetic surgical valves: insights from the VIVID registry. Eur Heart J. (2018) 39:687–95. 10.1093/eurheartj/ehx455 29020413

[147] Gurvitch R Cheung A Bedogni F Webb JG. Coronary obstruction following transcatheter aortic valve-in-valve implantation for failed surgical bioprostheses. Catheter Cardiovasc Interv. (2011) 77:439–44. 10.1002/ccd.22861 21328685

[148] Sultan I Siki M Wallen T Szeto W Vallabhajosyula P. Management of coronary obstruction following transcatheter aortic valve replacement. J Card Surg. (2017) 32:777–81. 10.1111/jocs.13252 29143378

[149] Ribeiro HB Webb JG Makkar RR Cohen MG Kapadia SR Kodali S. Predictive factors, management, and clinical outcomes of coronary obstruction following transcatheter aortic valve implantation: insights from a large multicenter registry. J Am Coll Cardiol. (2013) 62:1552–62. 10.1016/j.jacc.2013.07.040 23954337

[150] Hamdan A Barbash I Schwammenthal E Segev A Kornowski R Assali A. Sex differences in aortic root and vascular anatomy in patients undergoing transcatheter aortic valve implantation: a computed-tomographic study. J Cardiovasc Comput Tomogr. (2017) 11:87–96. 10.1016/j.jcct.2017.01.006 28139364

[151] Khan JM Greenbaum AB Babaliaros VC Rogers T Eng MH Paone G. The BASILICA trial: prospective multicenter investigation of intentional leaflet laceration to prevent TAVR coronary obstruction. JACC Cardiovasc Interv. (2019) 12:1240–52. 10.1016/j.jcin.2019.03.035 31202947 PMC6669893

[152] Khan JM Dvir D Greenbaum AB Babaliaros VC Rogers T Aldea G. Transcatheter laceration of aortic leaflets to prevent coronary obstruction during transcatheter aortic valve replacement: concept to first-in-human. JACC Cardiovasc Interv. (2018) 11:677–89. 10.1016/j.jcin.2018.01.247 29622147 PMC6309616

[153] Komatsu I Mackensen GB Aldea GS Reisman M Dvir D. Bioprosthetic or native aortic scallop intentional laceration to prevent iatrogenic coronary artery obstruction. Part 1: how to evaluate patients for BASILICA. Eurointervention. (2019) 15:47–54. 10.4244/EIJ-D-19-00057 30967362

[154] Komatsu I Mackensen GB Aldea GS Reisman M Dvir D. Bioprosthetic or native aortic scallop intentional laceration to prevent iatrogenic coronary artery obstruction. Part 2: how to perform BASILICA. Eurointervention. (2019) 15:55-66. 10.4244/EIJ-D-19-00056 30888958

[155] Dvir D Khan J Kornowski R Komatsu I Chatriwalla A Mackenson GB. Novel strategies in aortic valve-in-valve therapy including bioprosthetic valve fracture and BASILICA. Eurointervention. (2018) 14:AB74–AB82. 10.4244/EIJ-D-18-00667 30158098

[156] Mayo RP Yaakobovich H Finkelstein A Shadden SC Marom G. Impact of BASILICA on the thrombogenicity potential of valve-in-valve implantations. J Biomech. (2021) 118:110309. 10.1016/j.jbiomech.2021.110309 33601181

[157] Khodaee F Qiu D Dvir D Azadani AN. Reducing the risk of leaflet thrombosis in transcatheter aortic valve-in-valve implantation by BASILICA: a computational simulation study. Eurointervention. (2019) 15:67–70. 10.4244/EIJ-D-19-00048 30888960

[158] Hatoum H Maureira P Lilly S Dasi LP. Impact of leaflet laceration on transcatheter aortic valve-in-valve washout: BASILICA to solve neosinus and sinus stasis. JACC Cardiovasc Interv. (2019) 12:1229–37. 10.1016/j.jcin.2019.04.013 31272669 PMC6613808

[159] Rayz V Boussel L Ge L Leach J Martin A Lawton M. Flow residence time and regions of intraluminal thrombus deposition in intracranial aneurysms. Ann Biomed Eng. (2010) 38:3058–69. 10.1007/s10439-010-0065-8 20499185 PMC2940011

[160] Gorbet MB Sefton MV. Biomaterial-associated thrombosis: roles of coagulation factors, complement, platelets and leukocytes. Biomaterials. (2004) 25:219–41. 10.1016/B978-008045154-1.50025-3 15147815

